# Hepatitis C: A Rare Cause of Subacute Paralysis

**DOI:** 10.7759/cureus.39887

**Published:** 2023-06-02

**Authors:** Jennifer Ngo, Robert Andalon, Luis Delgado, Mariam Gilmore, Kelly Downey, Patrick Wu, Made Sutjita

**Affiliations:** 1 Internal Medicine, University of California Riverside School of Medicine, Riverside, USA; 2 School of Medicine, University of California Riverside, Riverside, USA; 3 Internal Medicine, Riverside University Health System Medical Center, Riverside, USA; 4 Infectious Disease, Riverside University Health System Medical Center, Riverside, USA

**Keywords:** incontinence, cryoglobulinemia, vasculitis, transverse myelitis, hepatitis c

## Abstract

The effects of the hepatitis C virus (HCV) on the nervous system have been primarily reported with a pathology of the peripheral nervous system through the involvement of a vasculitic process via cryoglobulinemia. A review of the recent literature reinforced the likely association between chronic HCV infection and transverse myelitis (TM), but the causal relationship remains elusive. Here, we present a rare case of acute TM developing over the course of days from symptom onset and a concomitant new diagnosis of HCV infection. A 31-year-old male with a medical history of stimulant use disorder with intravenous methamphetamine use presented to the hospital for acute bilateral leg weakness. The weakness was predominantly in his thighs and later progressed to his calves over the course of days. He denied urinary or fecal incontinence; however, on hospital day two, he developed acute urinary retention requiring the insertion of a Foley catheter. An initial MRI of the spine revealed an intramedullary T2 hyperintense signal at the lower thoracic cord concerning for TM, multiple sclerosis, ischemia, or neoplasm. MRI of the brain was unremarkable. Lumbar puncture results also displayed no abnormalities. HCV screening should be considered in all patients who develop acute neurological deficits that are not otherwise explained, such as TM, given the significant morbidity associated with delayed treatment.

## Introduction

The hepatitis C virus (HCV) is an enveloped, single-stranded, positive-sense RNA virus that can manifest as an acute or chronic infection. Approximately 58 million people worldwide had chronic HCV infection in 2019 and about an estimated 1.5 million people acquire HCV every year [1,2].

Although HCV is commonly known to primarily affect the liver in the form of cirrhosis, it can also present with extrahepatic manifestations such as cryoglobulinemia-induced vasculitis. This process is driven by the presence of numerous cold insoluble immunoglobulins (i.e., monoclonal or polyclonal IgM, IgG, IgA, and/or complement proteins) in the blood, their subsequent deposition, and inflammation of small-to-medium-sized blood vessels [3]. Traditionally, cryoglobulinemia has been linked to HCV-associated neurological manifestations in up to 54.3% of patients which include peripheral neuropathy, multiple mononeuritis, and polyradiculoneuropathy [4]. Involvement of the cerebral vessels in the form of occlusive vasculopathy and vasculitis with narrowing of cerebral arteries are theorized as plausible mechanisms for the ischemic strokes, transient ischemic attacks, and lacunar events seen in patients with HCV.

However, chronic HCV has also been theorized to induce immune-mediated inflammatory demyelination of the central nervous system to encompass presentations such as encephalitis and myelitis. Although the latter condition is more generally known as myelopathy from inflammation of the spinal cord through a demyelinating disease such as multiple sclerosis, infection was identified as the cause in 12% of cases in one study. Myelitis can present with motor, sensory, and autonomic dysfunction. It can lead to devastating degrees of residual disability if not treated promptly [5].

## Case presentation

**Figure 1:**
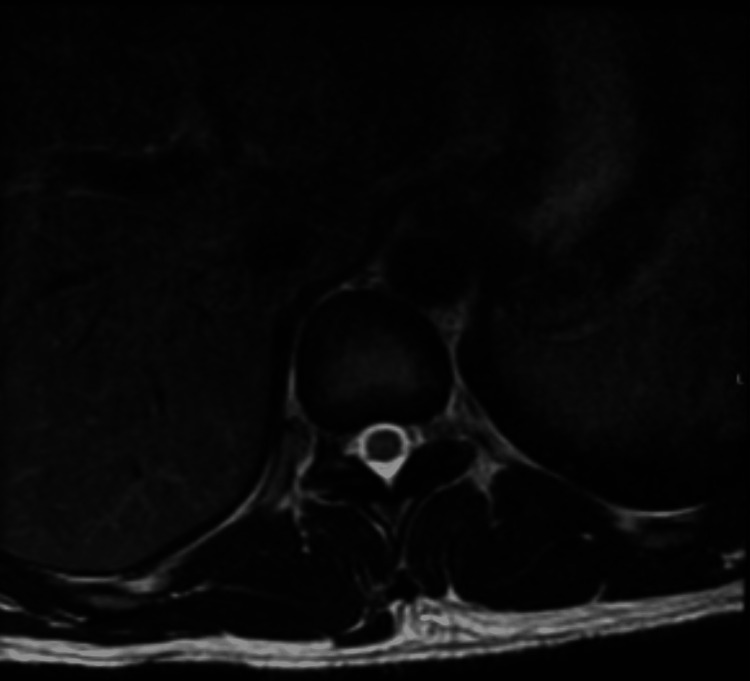
Thoracic spine MRI with contrast enhancement at the level of the conus medullaris on hospital day one showing unremarkable MRI.

**Figure 2:**
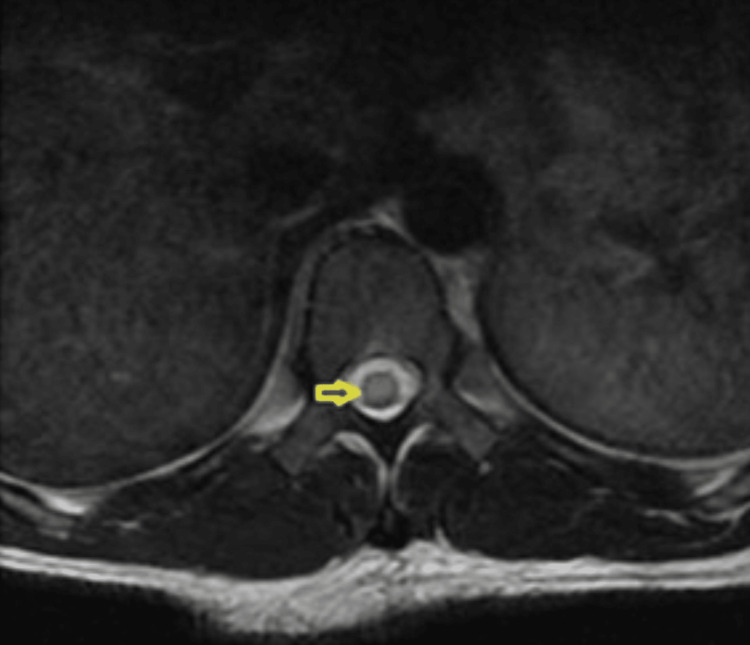
Repeat thoracic spine MRI on hospital day four with contrast enhancement showing an intramedullary T2 hyperintense signal and expansion at the lower thoracic cord/conus medullaris.

A 31-year-old right-handed male from California with a past medical history of right thigh abscess from a laceration sustained after a mechanical fall a few weeks before the current presentation, intravenous stimulant use disorder with methamphetamines, chronic paranoid-type schizophrenia, homelessness, and unemployment presented to the hospital for acute bilateral leg weakness. The weakness was predominantly in his thighs and later progressed to his calves over the course of days. He denied urinary or fecal incontinence. Vital signs upon admission were within normal limits. Physical examination was significant for flaccid bilateral lower extremities with absent patellar and Achilles reflexes. Upper extremity strength was 5/5 bilaterally. His gait was unable to be assessed. Initial contrast-enhanced T spine MRI on the day of admission was unremarkable, without findings to suspect epidural abscess or discitis/osteomyelitis (Figure 1). However, the L spine MRI visualized a central disc protrusion at L5-S1 with surrounding epidural enhancement that may represent granulation tissue in the setting of disc herniation or early epidural phlegmon. He was subsequently started on empiric cefepime, metronidazole, and vancomycin antibiotics. Brain MRI was unremarkable. On hospital day two, he developed acute urinary retention and required the insertion of a Foley catheter. Repeat contrast-enhanced T spine MRI on hospital day four showed intramedullary T2 hyperintense signal and expansion at the lower thoracic cord/conus medullaris concerning for transverse myelitis (TM), multiple sclerosis, or neoplasm (Figure 2). Per neurology recommendations, T spine MRI was repeated with diffusion-weighted imaging/apparent diffusion coefficient sequences on hospital day eight to rule out spinal infarct; however, the result was inconclusive for spinal cord infarct.

Subsequent CT of the abdomen and pelvis showed no liver lesion. Neurology service recommended a lumbar puncture. Lumbar puncture revealed no oligoclonal banding, no growth on culture, and normal glucose as well as protein level (Table 1). He was initially started on empiric antibiotics which were later discontinued when the cerebrospinal fluid (CSF) analysis revealed normal cell count, glucose, protein, and negative bacterial culture. Further investigation revealed negative rapid plasma reagin, quantiferon, cryptococcal screen, *Coccidioides* antibody, and HIV 1-2 Ag Ab screen. Autoimmune workup such as antinuclear antibody, rheumatoid factor, antineutrophilic cytoplasmic antibody, ganglioside GD1A, GD1B, GQ1B, GM1, and myelin oligodendrocyte glycoprotein antibodies were negative. Complement and anticardiolipin levels were unremarkable. HCV infection was confirmed with elevated viral load. Before this, the patient had no history of HCV. Additionally, serum cryoglobulins were found to be positive (Table 2).

**Table 1:** Cerebrospinal fluid analysis findings.

Test name	Results
*Coccidioides *Ab, CF	<1:2
*Cryptococcus *screen	Not detected
Glucose (mg/dL)	63
Protein (mg/dL)	44
Oligoclonal banding	No banding
Culture	No growth at 72 hours

**Table 2:** Relevant laboratory findings. ANA = antinuclear antibody; ANCA = antineutrophilic cytoplasmic antibody; HAV = hepatitis A antibody; HBc = hepatitis B core antigen; HBsAg = hepatitis B surface antigen; HCV = hepatitis C virus; WBC = white blood cell; RBC = red blood cell

Test name	Results
C-3 Complement (90.00–180.00 mg/dL)	116
C-4 Complement (10.00–40.00 mg/dL)	23.4
Cardiolipin Ab (IgA)	<11
Cardiolipin Ab (IgG)	<14
Cardiolipin Ab (IgM)	<12
ANA screen	Negative
ANCA screen	Negative
Myeloperoxidase antibody	<1.0
Proteinase-3 antibody	<1.0
QuantiFERON	Negative
HIV 1-2	Nonreactive
HAV IgM	Nonreactive
HBc IgM	Nonreactive
HBsAg	Nonreactive
HCV Ab	Reactive
HCV RNA, quantitative PCR (IU/mL)	1,130,000
HCV genotype	1a
Serum cryoglobulin	Positive
WBC (0–5/cumm)	8
RBC (0–4/cumm)	250
Monocyte % (94–100%)	9%
Neutrophil % (0–4%)	91%

A five-day course of 1 g intravenous (IV) methylprednisolone regimen for TM was initiated per rheumatology recommendation. Repeat neurological examination on the second day of corticosteroid treatment was significant for persistent urinary retention, bilateral lower extremity paralysis with areflexia, and decreased light touch sensation at the level of T10. Vibration and proprioception were intact. Afterward, the patient was switched to 60 mg prednisone PO for three days, then to 60 mg IV methylprednisolone BID for four days, and back to an increased dose of prednisone 80 mg PO daily.

After failure to improve with corticosteroids alone, he subsequently received plasma exchange therapy (PEX) for five days as well as a single administration of rituximab. After just one day of PEX, the patient was able to perform slight wiggling movements of his right foot. He then received additional PEX treatments every other day on three separate occasions along with an additional dose of rituximab while continuing the daily 80 mg prednisone PO daily. Before discharge from the hospital, the patient notably had improved strength in his right lower extremity. A prolonged corticosteroid taper was initiated, and he was subsequently discharged to a nursing facility with close follow-up with rheumatology and hepatology for possible HCV treatment on an outpatient basis.

## Discussion

The effects of HCV on the peripheral nervous system through a vasculitis process caused by cryoglobulinemia are well-documented. Furthermore, cryoglobulinemia has been linked to HCV-associated neurological manifestations in the form of peripheral neuropathy, multiple mononeuritis, and polyradiculoneuropathy. A review of the recent literature reinforced the likely association between chronic HCV infection and TM, but the causal relationship remains elusive.

TM is often an acute or subacute inflammatory process that manifests with spinal cord dysfunction, resulting in motor and sensory loss caudal to the spinal cord lesion. If no neurological disease or compression is present, the potential etiologies remain diverse and include infectious and rheumatological processes such as antiphospholipid syndrome, vitamin B12 deficiency, thrombosis or vasculitis at the watershed region of the artery of Adamkiewicz, and multiple sclerosis. Postulated theories behind the mechanism between HCV and TM include autoimmune-related triggers or direct myelitis due to HCV. Theories suggest that HCV manifestations similarly prime the immune system to form autoantibodies against the blood vessels and/or myelin [6-8]. Our suspicion for atherosclerotic spinal cord ischemia is low. The patient was relatively young at 31 years old and had no history of diabetes and atherosclerotic disease. However, cryoglobulinemia may cause vasculitis and indirectly cause spinal cord infarct and myelitis [9-12]. Amphetamine vasoconstriction injury of the spinal cord was on the differential; however, his urine drug screen was negative for amphetamine but positive for tetrahydrocannabinol. Bacterial pyogenic myelitis was less likely with normal cell count, glucose, and protein on CSF analysis. In addition, CSF and blood cultures drawn before the administration of antibiotics were negative. There have also been instances of Lyme neuroborreliosis-induced TM that could postulate an explanation for the patient’s deterioration [13]. In this instance, however, the patient had no history of recent travel outside of California, which is not endemic to Lyme disease. He also did not display any physical symptoms such as erythema chronicum migrans which is typical of the Lyme disease rash.

## Conclusions

We describe a rare case of TM associated with HCV infection. HCV screening should be considered in all patients who develop acute neurological deficits that are not otherwise explained, such as TM, given the significant morbidity associated with delayed treatment.
